# Effect of Specific Acupuncture Therapy Combined with Rehabilitation Training on Incomplete Spinal Cord Injury: A Randomized Clinical Trial

**DOI:** 10.1155/2021/5671998

**Published:** 2021-12-26

**Authors:** Feng Xiong, Jingkang Lu, Hongxia Pan, Fengyi Wang, Yaqin Huang, Yiwei Liu, Lingxin Li, Rengang Zhang, Yulong Wang, Chengqi He, Wei Quan

**Affiliations:** ^1^Department of Rehabilitation Medicine Center and Institute of Rehabilitation Medicine, West China Hospital, Sichuan University, Chengdu, Sichuan, China; ^2^Key Laboratory of Rehabilitation Medicine in Sichuan Province, Chengdu, Sichuan, China; ^3^Department of Rehabilitation, Shenzhen Second People's Hospital, The First Affiliated Hospital of Shenzhen University, Shenzhen, China

## Abstract

Acupuncture therapies were used to treat spinal cord injury (SCI) and its complications. To assess the effect of a specific acupuncture therapy combined with rehabilitation training for inpatients with incomplete SCI, we conducted an assessor-blinded, randomized controlled clinical trial in the Department of Rehabilitation Medicine Center in West China Hospital, Sichuan University. Seventy-two participants diagnosed with incomplete SCI were randomly assigned into 3 groups of 24 patients each, with data collection completed in December, 2019. Participants were randomly assigned (1 : 1 : 1) to 3 groups to receive treatment for 4 weeks, 5 times/week of acupuncture for Continuous Acupuncture Treatment (CAT) group, 3 times/week for Intermittent Acupuncture Treatment (IAT) group, and no acupuncture for Control group; all 3 groups received routine rehabilitation training. The primary outcome was the change of American Spinal Injury Association (ASIA) motor score from baseline to week 4. Secondary outcomes included sensory score, Modified Barthel Index (MBI). At week 4, CAT group had a higher motor score and MBI score increase than the control group (mean difference 10.52, 17.36; *p* *<* 0.001, *p* *<* 0.01, respectively). CAT group had more increase in motor score and MBI than IAT group (mean difference 5.55, 14.77; *p* *<* 0.05, *p* *<* 0.05, respectively). But the difference among groups in the increase of sensory score was not statistically significant. Acupuncture resulted in a higher motor score and MBI after 4 weeks. And the dosage of 5/week led to more improvement in motor score and MBI than that of 3/week. The results suggested that a dosage of 5/week of acupuncture is safe and more effective for SCI than 3/week. But further research is needed to determine the best intervention dosage, long-term efficacy, and underlying mechanism. This trial is registered with ChiCTR1900021530.

## 1. Introduction

Spinal cord injury (SCI) is a neurological condition that is common in the clinic but is often severe; most of the patients ended up with quadriplegia or paraplegia. At present, rehabilitation treatments for SCI have been proven to be effective [1], but the effects are often slow and limited [2], so it is necessary to seek better rehabilitation treatments.

Acupuncture has a wide range of applications, and acupuncture treatments for SCI have been studied across the world, but there have been only a few randomized controlled trials reporting treatment effects on neural function (motor and sensory) in English [3–6]. Most of the existing literature regarding the clinical effect of acupuncture on SCI only reported other outcomes such as pain scale or molecular indicators [3–9]. At present, the acupoints used in most of the studies investigating acupuncture treatment for SCI were mainly on the abdomen, the back, and the lower extremities [3–6]. Acupuncture relies on mobilizing the energy within the human body itself, but because of the stagnation of Qi and blood below the damaged segment after SCI, it is difficult to obtain a satisfactory result using treatments consisting mostly of acupoints below the injured segment.

Besides the location and number of acupoints, the dosage of acupuncture for SCI also varied between centers. Some of the common dosages are three times, five times, or seven times per week. Up until now, there has not been a trial investigating the dosage of acupuncture treatments for SCI.

To help solve the problem of dosage, this study aimed to investigate the intervention frequency of acupuncture therapy on incomplete SCI.

## 2. Methods

**Figure 1:**
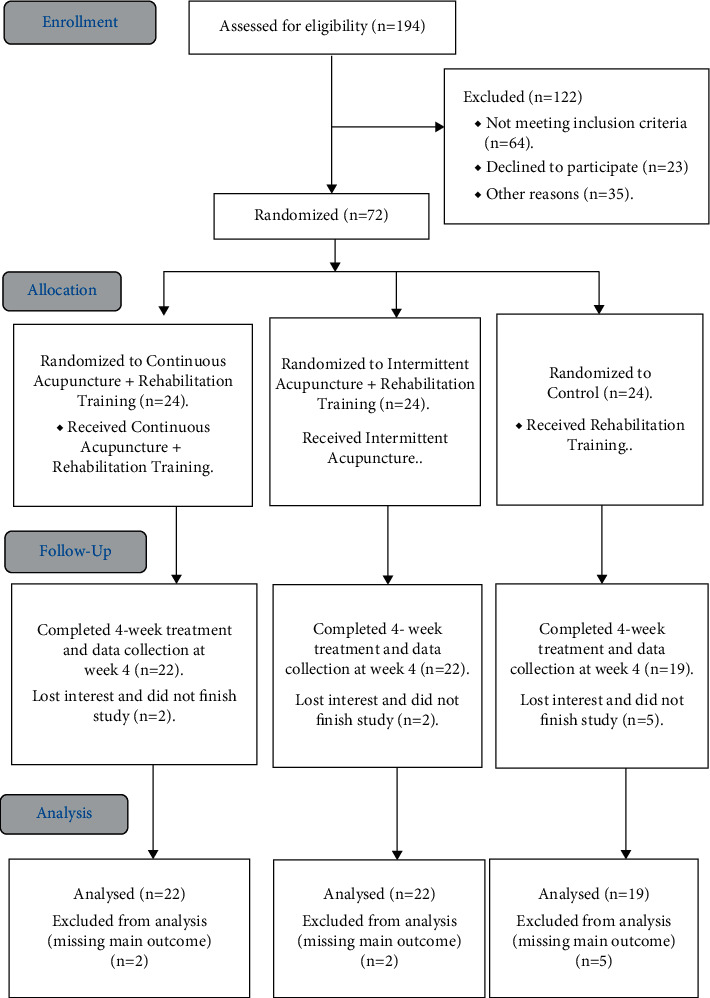
Consolidated Standards Of Reporting Trials (CONSORT) 2010.

This assessor-blinded, randomized controlled clinical study was conducted in West China Hospital in Sichuan, China. The medical ethical committee of West China Hospital, Sichuan University, approved the study protocol (no. 2018-253). The trial was registered at the Chinese Clinical Trial Registry (ChiCTR1900021530). This study was supported by the National Natural Science Foundation of China (grant no. 81572231). Written informed consent was obtained from all participants. The study process was shown in Figure 1.

### 2.1. Participants

Patients with SCI were recruited from inpatients at the Department of Rehabilitation Medicine, West China Hospital, Sichuan University, between August, 2018, and December, 2019. Interested patients were initially screened according to the inclusion and exclusion criteria at the neural rehabilitation ward. Participants were not compensated for study participation. Participants' self-reported ethnicity information was routinely collected without ethnic discrimination.

### 2.2. Inclusion Criteria

The inclusion criteria were as follows: age 18 to 75 years, a clear history of trauma by surgery or accidents of the spinal cord, diagnosis with SCI by magnetic resonance imaging and clinical symptoms, classification as grade C or D according to the American Spinal Injury Association (ASIA) 2011 classification of SCI, passing the shock phase of SCI, already went through surgery (if needed), stable vital signs, clear consciousness, the liability to cooperate with the treatments and tests and answer the questionnaire, and agreement to participate and sign informed consent.

### 2.3. Exclusion Criteria

The exclusion criteria included: shock phase of SCI, complete SCI, inability to complete the sessions due to various reasons, severe deep vein thrombosis, open fractures, infection, severe osteoporosis, and other complications that may prevent the patients from participating in the rehabilitation sessions; heart failure, cardiovascular and cerebrovascular accidents within 1 year, malignant tumors, and other serious diseases that may lead to interruption of the experiment or the observation of outcome indicators; and skin infections around the acupuncture points, history of severe fainting during acupuncture, and other conditions that may prevent the patients from receiving acupuncture.

### 2.4. Randomization and Blinding

Seventy-two participants were randomly assigned to 3 groups according to the random sequences generated using Excel software. The random sequences were each sealed in envelopes and were not to be opened until each patient's enrollment. The 3 groups were CAT group, IAT group, and the control group, with ratio 1 : 1 : 1, 24 participants in each group. The outcome assessors, therapists (rehabilitation therapists other than an acupuncturist), and statisticians were blinded to treatment allocation. Acupuncturists were not blinded due to the nature of the intervention. Acupuncturists, other therapists, outcome assessors, and statisticians are four separate groups of people.

### 2.5. Intervention

Hwato brand disposable acupuncture needles (size 0.25 × 40 mm, or 0.25 × 75 mm, manufactured by Wuxi Jiajian Medical Devices Co., Ltd) were used.

#### 2.5.1. Routine Rehabilitation

All three groups of patients were treated with routine rehabilitation training, and the necessary symptomatic treatment (for example, catheterization) was performed for complications. Both CAT group and IAT group received acupuncture treatments: 5/week (Monday to Friday) for CAT group and 3/week (Monday, Wednesday, and Friday) for IAT group. The treatments lasted for 4 weeks.

All included patients received routine rehabilitation training. It mainly included passive training, upright bed treatment, active training, sitting balance, wheelchair shift, the activity of daily life training, and counseling of patients and their families.

For patients with neurogenic bladder, intermittent catheterization was also required. The urine was drained from the bladder according to the bladder sensation or a timetable using a catheter. A drinking-catheterization plan was made for each patient before the experiment.

#### 2.5.2. Acupuncture Treatments

Besides the rehabilitation treatment mentioned above, the acupuncture groups (CAT group and IAT group) shall receive acupuncture treatment.

Acupuncture treatment began on the first Monday after enrollment. The selected area should be disinfected with 75% alcohol, and the doctor's hand should be disinfected by washing with soap or quick-drying disinfectant. To prevent fainting, patients should be prevented from exercising vigorously within 1 hour before treatment, avoid acupuncture when hungry, and properly communicate with and comfort the patient to avoid mental stress. The patient was placed in a supine position during treatment exposing to the skin at the corresponding location. The room temperature was adjusted, and an infrared heater was used to maintain the surface body temperature of the patient if necessary.

*Baihui* (GV20) 0.5 to 0.8 cun (about 15 degrees angle with the skin, needle tip pointing to the lower back), bilateral *Hegu* (LI4) 0.5 to 0.8 cun (the palm in a relaxed position), bilateral *Wangu* (SI4) 0.3 to 0.5 cun, bilateral *Houxi* (SI3) 0.5 to 1.0 cun, bilateral *Yemen* (TE2) 0.3 ∼ 0.5 cun, bilateral *Zhigou* (SJ6) 0.5 to 1cun, bilateral *Shaofu* (HT8) 0.3 ∼ 0.5 cun, bilateral *Quchi* (LI11) 1.0 to 2.5 cun, bilateral *Jianyu* (LI15) (the tip of the needle points to the direction of *Jiquan* [HT1], 2 to 3 cun deep), and *Shousanli* (LI10) 1 to 2 cun [10] were applied (for all the above acupoints, the needle is inserted perpendicularly into the skin, if not specifically instructed otherwise).

Ren meridian acupoints: [11] the position opposite to the injury point, for example, the lumbosacral region, corresponds to the upper chest, and the upper part of the thoracic spinal cord corresponds to the lower abdomen. The specific correspondence of the acupoints to the injured segments is shown in Table 1. And the cervical segments correspond to the *Houxi* (SI3) and the *Shenmai* (BL62).

After needle insertion, small manipulations of twirling were performed on the needles to reach de qi sensation, which is believed to be essential for acupuncture efficacy [12]. Each session lasted 30 minutes and then the needles were carefully removed and counted.

The depth of acupuncture is measured by the unit “cun,” which is the width of the thumb joint of the patient in most cases.

The specific location of all the acupoints mentioned above was determined according to WHO standard acupuncture point locations in the Western Pacific region (Manila: World Health Organization; 2008). And all acupuncture procedures were performed by professional acupuncturists with years of experience.

Participants in the rehabilitation group received only routine rehabilitation.

Throughout the trial, aside from the acupuncture intervention, the participants were treated the same as nonparticipants, and proper treatments (medicine or therapies) were given for SCI-related complications.

### 2.6. Outcome Measures

The main outcome was a change of motor score from baseline to week 4 (28 days).

The secondary outcome included sensory score, light touch (LT) and pinprick (PP), and Modified Barthel Index (MBI).

Adverse events were documented throughout the trial.

Based on their potential association with the acupuncture needling procedure, adverse events were categorized by acupuncturists and related specialists as treatment-related or non–treatment-related within 24 hours of occurrence.

## 3. Statistical Analysis

Data were presented as mean ± standard deviation (SD). The statistical analysis was conducted using SPSS software version 20. The comparisons of continuous variables between treatment groups were assessed using the *t*-test. The primary outcome was analyzed according to the intention-to-treat principle. The change from baseline in the motor score at week 4 was analyzed by fitting a mixed-effect model using baseline value as a covariate, treatment as a fixed effect, and interaction between treatments as random effects accounting for acupuncturist differences. Kappa analysis was used to analyze the distribution of the injured segment.

## 4. Results

Among the 72 randomized participants (mean [SD] age, 43.36 [14.43] years), 63 completed the study and 9 withdrew from the study due to reasons unrelated to the study (2 in CAT group, 2 in the IAT group, and 5 in the control group).

No statistical differences were found in the age and injured segment (Table 2).

The mean motor score at baseline was 59.91 for the CAT group, 57.86 for the IAT group, and 60.42 for the control group (no statistical difference between the 3 groups, *p* *>* 0.05) (Table 2). At week 4, the CAT group (15.05) had a greater increase in motor score than the IAT group, and the IAT group had a greater increase (9.50) than the control group: mean differences were 10.52 (95% CI, 6.14 to 14.90; *p* *<* 0.001) between CAT group (15.05) and the control group (4.53), 4.97 (95% CI, 0.43 to 9.52; *p* *<* 0.05) between the IAT group (9.50) and the control group (4.53), and 5.55 (95% CI, 0.32 to 10.77; *p* *<* 0.05) between the CAT group (15.05) and the IAT group (9.50) (Table 3).

The mean LT score at baseline was 81.19 for the CAT group, 71.70 for the IAT group, and 68.00 for the control group (no statistical difference between each group, *p* *>* 0.05) (Table 2). At week 4, the difference in the increase of LT score between each two of the three groups was not statistically significant: mean differences were 0.23 (95% CI, −11.05 to 10.60; *p* *>* 0.05) between CAT group (7.95) and the control group (8.18), 1.02 (95% CI, −10.63 to 12.68; *p* *>* 0.05) between IAT group (9.20) and the control group (8.18), and 1.25 (95% CI, −9.47 to 6.97; *p* *>* 0.05) between CAT group (7.95) and IAT group (9.20) (Table 3).

The mean PP score at baseline was 74.76 for the CAT group, 69.60 for the IAT group, and 62.82 for the control group (no statistical difference between each group, *p* *>* 0.05) (Table 2). At week 4, the difference in the increase of PP score between each two of the three groups was not statistically significant: the mean difference was 3.84 (95% CI, −16.05 to 8.37; *p* *>* 0.05) between the CAT group (8.33) and control group (11.41), 3.61 (95% CI, −16.56 to 9.34; *p* *>* 0.05) between the IAT group (7.80) and the control group (11.41), and −0.23 (95% CI, −9.97 to 9.51; *p* *>* 0.05) between CAT group (8.33) and IAT group (7.80) (Table 3).

The mean MBI at baseline was 18.87 for the CAT group, 32.22 for the IAT group, and 28.29 for the control group (no statistical difference between each group, *p* *>* 0.05) (Table 2). At week 4, the CAT group had a greater increase in MBI (33.52) than the control group (16.17), and the CAT group (33.52) had more increase than the IAT group (18.75). But the difference between the IAT group (18.75) and the control group (16.17) in the increase of MBI was not statistically significant: mean differences were 17.36 (95% CI, 5.92 to 28.79; *p* *<* 0.01) between the CAT group (33.52) and the control group (16.17), 2.58 (95% CI, −7.83 to 13.00; *p* *>* 0.05) between the IAT group (18.75) and the control group (16.17), and 14.77 (95% CI, 3.24 to 26.30; *p* *<* 0.05) between the CAT group (33.52) and the IAT group (18.75) (Table 3).

No treatment-related severe adverse events (such as acupuncture fainting that persisted after the acupuncture procedure, severe pain or infection around acupoints, bleeding of more than 1 mL of the acupoints after acupuncture, and damage of major nerve or vessels around the acupoints) were reported during the trial. And no severe advancements of SCI-related complications (such as neurogenic bladder, neurogenic bowel, and pressure sores) were found during the trial.

## 5. Discussion

The purpose of this randomized controlled trial is to determine the ideal dosage of acupuncture therapy combined with rehabilitation training for SCI in improving neural function and daily living ability.

Acupuncture improved ASIA Motor Score and Modified Barthel Index in patients with SCI and seemed to have no effect in improving the sensory score in this study.

The acupuncture therapy did not result in a better outcome in the sensory score. It could be that this specific acupuncture therapy does not have enough effect in improving sensory function or the sample size is still limited, or the frequent needling may have built up the patients' tolerance for mild stimulus.

Significant differences in the motor score and MBI were observed between the CAT group and IAT group, meaning that increased dosage of acupuncture treatment led to more improvement in motor function and daily living ability; the dosage of 5/week can be considered as safe and more effective.

The IAT group did not show improvement in motor, sensory, and MBI score, meaning that intermittent therapy (3/week) may not have enough effect on patients with SCI to show a statistical difference, or the IAT lacked stimulus to start certain molecular pathways which existed in CAT group, or the sample size was not big enough to show statistical difference.

Acupuncture has not been used as a routine treatment for SCI, except in several Asian countries [4]. Most of the previous studies emphasized acupuncture treatment for complications of SCI such as neurogenic bladder, while less attention was paid to the motor and sensory function or daily living ability. Previous randomized clinical trials investigating acupuncture for SCI reported a dosage of once per day [13–17], 6/week [13, 18], or 5/week [11, 19], but no trials have done the comparison between different dosages [20].

Skilled acupuncturists design useful acupuncture treatment plans by following ancient wisdom but lacking modern scientific interpretation. Take dosage, for example, the underlying mechanisms of it, what dosage works best and why, may just be the key to deciphering the secrets in Chinese Traditional Medicine.

The Bian Que needle method which was used to select acupoints in this study is from the Classical Acupuncture method, one of the four famous methods of Taiwan acupuncture, and was inherited from the acupuncture masters Zuoyu Zhou and Peirong Sun. [21] Classical Acupuncture is specialized in midnight-noon ebb-flow, Linggui Bafa, Wumen Shibian method, Bian Que needle, et cetera [21]. And its effectiveness on a few other diseases has been studied and proved [22, 23].

Admittedly, it would be better that the sample size of this study was bigger. Due to the limited number of participants, this study has not been able to analyze certain outcome measures and if acupuncture affects them. And the study did not contain a long time follow-up to study long-term effects. And since all the participants were Chinese thus not easily fooled by sham acupuncture and the acupoints used in this study were not easy to conceal, we did not design a sham acupuncture group. And due to the popularity of acupuncture among Chinese residents, there might have been a placebo effect on the two acupuncture groups which may have affected the results.

The strengths of this study are as follows. The dosage of acupuncture treatment for SCI had not been studied in any previous trials investigating SCI; it is the first time that the topic has been properly studied. And the sample size is relatively large compared with previous randomized controlled trials studying acupuncture for SCI, [3, 4] and the results showed that acupuncture is an effective and safe treatment and that the treatment frequency of 5/per week is better than 3/week. This result may be useful for clinical practice and further research.

Although we did conclude the more effective dosage of acupuncture for SCI, it may not be the best dosage there is; the mechanisms beneath it and whether it applies to other illnesses are still unknown.

## 6. Conclusion

In conclusion, based on the findings of this clinical trial, the recommended dosage of acupuncture for SCI is 5/week. We hope there will be more related studies in the future, to allow a deeper understanding of acupuncture.

## Figures and Tables

**Table 1:** The acupoints on the Ren meridian corresponding to segments of the spinal cord.

Spinal cord segment	Acupoints	Spinal cord segment	Acupoints
T1	*Qugu* (RN2), *Zhongji* (RN3)	T10	*Xiawan* (RN10), *Jianli* (RN11), *Zhongwan* (RN12)
T2	*Qugu* (RN2), *Zhongji* (RN3), *Guanyuan* (RN4)	T11	*Jianli* (RN11), *Zhongwan* (RN12)
T3	*Zhongji* (RN3), *Guanyuan* (RN4)	T12	*Zhongwan* (RN12), *Shangwan* (RN13)
T4	*Zhongji* (RN3), *Guanyuan* (RN4), *Shimen* (RN5)	L1	*Shangwan* (RN13), *Juque* (RN14)
T5	*Guanyuan* (RN4), *Shimen* (RN5), *Yinjiao* (RN7)	L2	*Shangwan* (RN13), *Juque* (RN14), *Zhenwei* (RN15)
T6	*Shimen* (RN5), *Yinjiao* (RN7), *Shenque* (RN8)	L3	*Juque* (RN14), *Zhenwei* (RN15)
T7	*Yinjiao* (RN7), *Shenque* (RN8), *Shuifen* (RN9)	L4	*Zhenwei* (RN15), *Zhongting* (RN16), *Danzhong* (RN17)
T8	*Shenque* (RN8), *Shuifen* (RN9), *Xiawan* (RN10)	L5	*Zhongting* (RN16), *Danzhong* (RN17), *Yutang* (RN18)
T9	*Xiawan* (RN10), *Jianli* (RN11)	S1	*Danzhong* (RN17), *Yutang* (RN18), *Zigong* (RN19)

**Table 2:** Data of the Control, IAT, and CAT groups at baseline.

Group	Control	IAT group	CAT group	*P*
Size	*N* = 24	*N* = 24	*N* = 24	
Age^*∗*^	44.00 (14.10)	44.17 (12.71)	41.92 (16.70)	0.257
Injured segment				
Cervical	9	8	8	0.976, (Likelihood ratio = 0.444)
Thoracic	9	9	8	
Lumbar	6	7	8	
Initial motor score^+^	60.42 (26.70)	57.86 (18.77)	59.91 (18.66)	0.255
Initial MBI score^+^	28.29 (27.08)	32.22 (26.44)	18.87 (19.99)	0.360
Initial LT score^+^	68.00 (29.14)	71.70 (25.98)	81.19 (21.10)	0.282
Initial PP score^+^	62.82 (30.48)	69.60 (29.35)	74.00 (24.59)	0.272

^*∗*^Data were presented as mean (SD). SD = standard mean difference; MBI = Modified Barthel Index; LT = light touch; PP = Pinprick; IAT = intermittent acupuncture treatment; CAT = continuous acupuncture treatment group.

**Table 3:** Outcome data of the control, IAT, and CAT groups at 4 weeks.

Group	Control group	IAT group	CAT group	*P*		
N	19	22	22	CAT vs. control	IAT vs. control	CAT vs. IAT
MS	64.95 (26.42)	67.36 (22.06)	74.95 (17.87)			
Change^+^	4.53 (4.62)	9.50 (8.79)	15.05 (8.39)	<0.001	0.160	0.038
MBI	44.46 (30.00)	49.63 (30.05)	52.39 (22.65)			
Change^+^	16.17 (17.75)	18.75 (18.10)	33.52 (20.93)	0.004	0.620	0.013
LT score	76.18 (24.95)	80.90 (24.21)	89.14 (20.26)			
Change^+^	8.18 (19.26)	9.20 (14.64)	7.95 (10.92)	0.966	0.859	0.760
PP score	74.24 (28.44)	77.40 (23.91)	82.33 (22.43)			
Change^+^	11.41 (21.13)	7.80 (16.73)	8.33 (13.83)	0.609	0.573	0.912

Data were presented as mean (SD). ^+^The change from baseline to 4 weeks. SD = standard mean; MS = Mortar Score; MBI = Modified Barthel Index; LT = Light touch; PP = Pinprick; IAT = intermittent acupuncture treatment; CAT = continuous acupuncture treatment group.

## Data Availability

The data used to support the findings of this study are available from the corresponding author upon request. .

## Supplementary Materials

Supplementary Materials Supplement File: STRICTA (Standards for Reporting Interventions in Clinical Trials of Acupuncture) Checklist for this study. .
